# Interdisciplinary communication of infectious disease research – translating complex epidemiological findings into understandable messages for village chicken farmers in Myanmar

**DOI:** 10.1186/2193-1801-3-726

**Published:** 2014-12-11

**Authors:** Joerg Henning, Than Hla, Joanne Meers

**Affiliations:** School of Veterinary Science, The University of Queensland, Gatton, Queensland 4343 Australia; Myanmar Livestock Federation, Bayint Naung Rd, Gyogon, Insein Tsp, Yangon, Myanmar

**Keywords:** Communication, Extension, Disease control, Myanmar, Village chicken, Newcastle disease

## Abstract

Improvement in animal disease control and prevention is dependent on several factors including farmers’ uptake of new technologies and skills, particularly in developing countries. Extension is the means by which information about these technologies and skills is delivered to farmers, in order that they can use this knowledge to improve farming practices and their quality of life. This implies a shift from traditional methods to new science-based methods of production. However, in many developing countries farmers are illiterate and unable to understand written outcomes of scientific research. This paper summarizes approaches to communicate epidemiological findings and reports on experiences obtained from a research project in Myanmar, where results from epidemiological field investigations and intervention studies were ‘translated’ in an understandable manner to village communities. Rural chicken farmers were the central focus of this extension work and simple and sustainable methods to improve the health and production of scavenging chicken flocks were promoted. Unique extension materials transformed scientific outputs published in international journals into clear pictographic messages comprehendible by villagers, while maintaining country-specific, traditional, religious and public perspectives. Benefits, difficulties and pitfalls in using extension methods to communicate advice on preventive veterinary medicine measures in different cross-cultural settings are discussed and guidelines on how to distribute epidemiological research results to illiterate farmers are provided.

**Electronic supplementary material:**

The online version of this article (doi:10.1186/2193-1801-3-726) contains supplementary material, which is available to authorized users.

## Background

Epidemiological field studies in developing countries are often exciting for researchers because infectious diseases are relatively common in these countries, and the diseases typically have severe impacts. Therefore many opportunities exist for improving animal health and for setting-up preventive measures. Usually in such studies, the animal health situation is described, analysed and interventions are tested in the field and then the outcomes of the research are published in scientific journals. The researcher receives the glory and adds the scientific achievements to his/her academic or research resume. Unfortunately most of the farmers who provided the information or participated in the field trials are never informed about the outcomes of the study, nor trained to incorporate the study results into their daily farming practice. Although the farmers might be mentioned in the acknowledgements section of the scientific paper, they gain little benefit from their participation in the study. Hence, the benefits of the research are often accrued by the researchers rather than the farmers who participated.

There are a variety of reasons why researchers ‘forget’ these farmers after the data have been gathered. The researcher might be already involved in another project or it may be beyond the scope of the funding and time limits of the research project to communicate the research findings back to the farmers. Furthermore the human ethics approval process for a research study conducted with farmers usually does not require that feedback on the study results has to be provided to the participating farmers. In addition, scientific research outcomes that are based on statistical models and that are presented in tables, figures or equations are difficult to understand by the non-scientist, let alone by farmers in developing countries who may be illiterate or have a poor education.

Preventive veterinary extension can be defined as practical and understandable advice given to individuals, groups or communities about livestock diseases, their prevention and treatment and about ways that influence the well-being, health and productivity of both humans and animals (McCrindle 1995). Extension is an ongoing process of getting useful information to people (communication) and then assisting those people to acquire the necessary knowledge, skills and attitudes to effectively utilize this information or technology (education) (Swanson and Claar 1984). The goal of the extension process is to enable people to use the skills, knowledge and information to improve their quality of life (Swanson et al. 1997). Extension methods include 1) mass communication methods, for example via radio, television, posters, leaflets and newspapers, 2) individualised methods where the farmer gets the full attention of the communicator, for example through farm visits, phone calls and letters and 3) group methods, for example group meetings, demonstrations, field days and tours (Oakley and Garforth 1985).

Epidemiological field research basically provides two types of outcomes that can be communicated to farmers: 1) observational studies that provide insights into prevalence and incidence of diseases and on risk factors of farming practices that influence disease occurrence and 2) intervention studies that evaluate methods that are more economically viable or that prevent the occurrence of disease or increase livestock production parameters. The latter field research is more applicable to farmers as solutions to overcome production or animal health constraints are tested under field conditions in active engagement with farmers. Thus farmers are able to experience how new interventions can influence their current farm management practices and other farmers not involved in the field research will observe and might adopt these interventions if they consider them as being valuable. On the other hand observational studies provide essential information to design intervention studies, but the seasonal patterns of disease frequency are probably already known to farmers and risk factors identified might perhaps not directly apply to farm management practices (e.g. climate, environmental or population density factors increasing disease frequency) while other factors might be difficult to implement on a farm level (e.g. preventing wild bird contact to scavenging village chickens to reduce the risk of Highly Pathogenic Avian Influenza or Newcastle disease outbreak occurrence).

Chickens are the most numerous livestock species in the world (Chemnitz and Becheva 2014). In most developing countries, small-scale producers are the dominant producer of chicken products: for example in Bangladesh, 98% of chickens and eggs come from small-scale farmers, in Ethiopia the proportion is 99% (Chemnitz and Becheva 2014). However, in many of these developing countries small-scale producers are poor and traditionally disadvantaged often with high levels of illiteracy or limited education. For these communities the concept of disease causation and its subsequent procedures of control and prevention of disease are difficult to comprehend (Alders et al. 2010).

We report here on various methods to communicate epidemiological research findings to illiterate or poorly-educated farmers in Myanmar and to advise these farmers on methods to improve village chicken health and production (Henning et al. 2007, 2008, 2009, 2013). This paper advocates the value of providing timely, relevant and accessible feedback from formal research activities. Although this work was conducted in a low income country, the underlying principles and approaches described here are equally applicable to the interaction between researchers and farmers in higher income settings.

## Methods

Between 2003 and 2007 a research project was conducted to improve village poultry productivity in Myanmar by identifying major constraints to village chicken production and then providing villagers with solutions to overcome these production-limiting factors. The project was funded by the Australian Centre for International Agricultural Research and was conducted by the University of Queensland, Australia in collaboration with the Ministry of Livestock and Fisheries, Livestock Breeding and Veterinary Department (LBVD), Myanmar. The project used a stepwise approach in conducting scientific investigations involving the village chicken-owning community that led to a participatory development of extension material and services to increase the awareness of smallholder poultry owners towards village chicken health and production.

Initially a series of epidemiological studies was conducted. A baseline survey indicated that mortality in young chicks and deaths from Newcastle disease were the two major constraints to chicken production followed by predation and mortality due to exposure to extreme weather conditions (Henning et al. 2007, 2008). Furthermore a serological survey of unvaccinated village chickens in 10 villages combined with on-farm recording of chicken deaths was used to describe the temporal pattern of Newcastle disease outbreak occurrence (Henning et al. 2008). Chicken mortalities with clinical signs of Newcastle disease, with temporal clustering of mortalities, and increases in Newcastle disease antibody titres in surviving birds in the same flock were considered to be caused by Newcastle disease virus. In some of the study flocks, tissue samples from dead birds were obtained and Newcastle disease was confirmed using PCR methods in the LBVD diagnostic laboratory (Henning et al. 2009).

These issues were then addressed in a 12-month intervention study with different interventions or treatments being applied to randomly selected flocks (Henning et al. 2009). In this study we compared the profitability for households of the use of Newcastle disease vaccination (Treatment 1 = TR1) with improved chick rearing management (TR3) to neither strategy (TR2) in an average sized flock of village chickens. Vaccination of individual birds against ND (TR1) was conducted using the thermostable live I-2 ND vaccine produced by LBVD. ND vaccine was administered in eye drops, by veterinarians from the Myanmar LBVD. Improved management of chicks (TR3) included the provision of both coops (for the protection of chicks from predation) and chick starter feed inside a creep feeder (to support chicks’ nutrition). The equipment for the confinement, the feeds, and the vaccinations were provided free of charge to participants during the randomised controlled trial, which was carried out from July 2004 until June 2005. Assuming costs to be incurred if farmers implement I-2 ND vaccination or improved chick management themselves we modelled the income from the sale of chickens under the three treatments and we were able to show that the group of farmers who introduced changes to the management of young chicks had an significant increase in the number of birds sold after a period of six months resulting in additional income from the sale of birds (Henning et al. 2009).

The focus of the last project phase was the dissemination of the study results and a training in preventive measures and this work is the focus of this paper. In discussions with LBVD staff and field veterinarians the following key messages on improving village chicken health and production were identified:

- Increasing farmers’ awareness towards the major causes of village chicken mortality
- Increasing farmers’ awareness towards the financial benefits of improved chick management
- Increasing farmers’ awareness on reducing the risk of mortality in village chickens by timely vaccination against Newcastle disease outbreaks using the I-2 vaccine

Local artists were employed to illustrate the extension messages. Extension messages were pilot-tested in group discussions with village chicken farmers and with individual farmers across three villages over a period of two weeks. The pilot-testing of the illustrations and images resulted in modifications of extension messages, mainly to incorporate local beliefs.

## Results

The extension messages produced in this phase of the project reflected specific scientific outcomes of the previous epidemiological field studies (Henning et al. 2007, 2008, 2009, 2013). Approximately 40 individual extension messages in form of illustrations were produced. In the following we provide examples of only three extension messages directly ‘translated’ from numerical results published in scientific publications.

**Figure 1:**
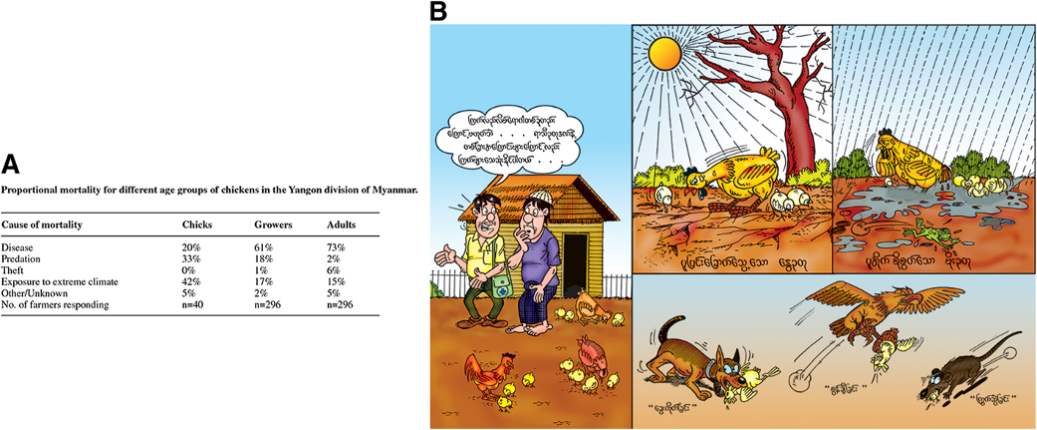
**Methods to summarize the main causes of village chicken mortality in Myanmar. A)** Proportional mortality rate of village chickens (Henning et al. 2007) and **B)** Extension cartoon illustrating main causes of mortality of village chickens.

Surveys on the main causes of mortality indicated that exposure to extreme weather conditions (heat in the dry season and rain during the monsoon season) and predation were the main cause of mortality in chicks (Figure 1A), with rats, dogs and birds of prey being the main predators (Henning et al. 2007). Contingency tables showing the proportional mortality rates for chicks were converted into pictures (Figure 1B) that were used in extension materials.

**Figure 2:**
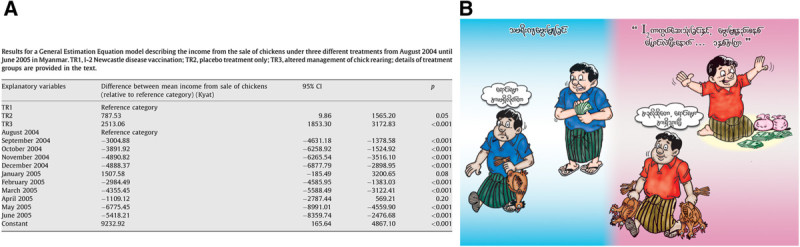
**Methods to demonstrate the increased income from the sale of chickens by adopting village chicken management interventions in Myanmar. A)** Results from a General Estimation Equation Model estimating income from the sale of chickens (Henning et al. 2009) and **B)** Extension cartoon showing the number of additional birds produced and additional money earned from the sale of chickens.

Results from a General Estimation Equation Model describing the income from the sale of chickens in the first year under the three different treatment strategies indicated that improved chick rearing provided an additional mean income from the sale of chickens of 2,513 Kyat per month (95% CI: 1,853, 3,172) compared to I-2 vaccination (Figure 2A) (Henning et al. 2009). Considering the market value of village chickens at the time of the project, this translated into selling up to five more birds per sale event per year (Figure 2B).

**Figure 3:**
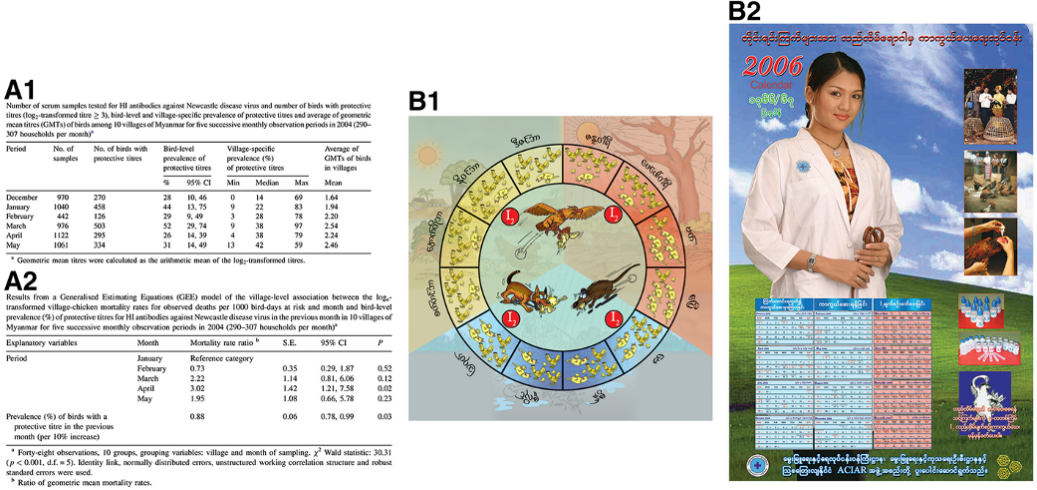
**Methods to describe the seasonal Newcastle disease occurrence in Myanmar. A1)** Bird and village level sero-prevalence of Newcastle disease antibodies over 6 months in village chickens (Henning et al. 2008) and **A2)** Results from a General Estimation Equation Model estimating village chicken mortality rates per month **B1)** Extension cartoons showing an annual cycle of mortality including the number of birds being alive or dying in a particular month and indicating months when Newcastle disease vaccination (I2) is required and **B2)** Newcastle disease vaccination calendar with a Myanmar actress posing as a veterinarian and the months when vaccination is required being highlighted (in red) (Henning et al. 2009).

A serological survey combined with an on-farm recording of flock mortalities indicated that Newcastle disease outbreaks predominately occur in the months of March and April (Figure 3A1 and Figure 3A2) (Henning et al. 2008). An annual chicken flock survival circle was developed showing individual months and the number of birds being alive or succumbing from disease in each month (Figure 3B1). The months when Newcastle disease vaccination using I-2 vaccine should be conducted to prevent these outbreaks are indicated with letters ‘I-2’.

Farmer workshops were the major tool to deliver the extension messages on improving village chicken health and on the use of the intervention methods. These workshops were conducted in common spiritual or communal places (e.g. in monasteries, in the house of the village head person, in schools or in village offices) from June until September 2007 in 47 villages in three different divisions of Myanmar (Yangon, Bago, Mandalay Divisions). Women and children as the main poultry keepers were the main target in the workshops. Training of trainers resulted in training of 14 field veterinarians and 47 village head people to deliver the extension messages on village chicken health and improved chick husbandry. Overall a total of 166 workshops were conducted with 3,969 trainees attending (representing about 50% of the 9,157 village households in the 47 villages). Farmers who had positive experiences with the interventions in the field studies were invited to report on their experiences. Farmers could purchase the extension equipment (bamboo coops, creep feeders, chick starter feed) used in the intervention studies. During the trainings sessions a total of 1750 complete sets of extension equipment for improved chick management were sold to farmers. Workshop participation was encouraged through lotteries in which participants could win some of the extension equipment. Village personnel who had significant association with poultry issues were identified to conduct these workshops in the future.

Extension messages comprising cartoons, diagrams and images were incorporated into the following extension materials:

- Flip charts with extension messages painted on strong, water proof canvas were used during farmer workshops. These charts presented the extension messages in sequence as a ‘theatre of messages’, they were ‘hands-on’ material and provided an entertainment to the audience. After the workshops the flip charts were further displayed in the schools of the villages.
- Booklets provided ‘take home’ messages and were handed out at the end of workshops. In addition to the cartoons, these booklets contained simple written statements and provided a readily available reference for the farmer. Vaccination calendars showing a famous Myanmar actress posing as a LBVD veterinarian (to encourage the display of the calendar in the farmers’ homes) and highlighting the months for I-2 vaccination were developed (Figure 3B2). This vaccination calendar was very popular and several editions had to be produced.
- Posters with extension messages were shown in central places such as major cross-roads or central locations, in or near the township veterinary offices, the house of the village head man or near shops or village markets.

## Discussion

Extension can be understood as providing a link between research and farmers. However, this only works if an appropriate method of communication is used. This project aimed to directly ‘translate’ scientific research outcomes in an understandable manner to the local village chicken owning community in Myanmar, and to use appropriate communication strategies. For example large, single-page calendars are often used as decoration in farm houses in Myanmar. We attracted farmers’ interest in the vaccination program by incorporating a photo of a famous Myanmar actress posing as a veterinarian in a calendar. Vaccination against Newcastle disease using I-2 vaccine is usually conducted three times per year, although the natural brooding in village flocks and the thereby continued introduction of susceptible young birds has to be considered in the vaccination scheme (Spradbrow 2014). Therefore LBVD of Myanmar recommended village chicken vaccinations four times per year and we incorporated this frequency in our extension messages.

General extension material on clinical signs of Newcastle disease and vaccination practices have been produced previously (Ahlers et al. 2009), but we developed extension messages that reflected specific published results obtained from our own village poultry research conducted in Myanmar. When preparing extension messages using cartoons or drawings, country-specific characteristics, religious and traditional beliefs have to be considered (Alders and Bagnol 2007). Extension messages developed for situations in other countries often do not work and have to be modified (e.g. poultry markets differ substantially between developed and less developed South East Asian countries). Furthermore pictures or cartoons should provide only single messages or limited information in local surroundings and ideally they should be colourful and entertaining. As with questionnaire development, pilot-testing of extension pictures and materials is an imperative. An important request made by farmers during the pilot testing of our images was that ‘bubbles of souls moving on’ should be incorporated into images showing dead birds, describing the goal of the Buddhist path that death is not the end of life, but the end of the body and the spirit will still remain.

The major tool to deliver our extension messages were village meetings. Other means of extension that we considered using in our project included radio, television and marionette theatre. In contrast to most African societies, where many local radio stations exist, radio transmission is not so common in Myanmar. Only government owned and controlled radio stations existed at the time of the project and Myanmar farmers were not accustomed to listening to radio programs. Therefore we evaluated that the use of radio as an extension method was not valuable. In contrast, television (TV) programs (two Myanmar government owned TV stations existed at the time) are often watched by village people in local tea shops or, if available, in the house of the TV owner in the village (although most villages had no or infrequent power supply) and could have been a useful medium for our extension messages. However, because ‘foreign’ involvement in TV advertisement at the time of the project was discouraged by government officials, we decided not to produce a TV advertisement on village chicken health.

Marionette theatre has a long tradition in Myanmar and Myanmar people have a great respect for an expert puppeteer. A puppet play would have been a useful tool to deliver a message on improving village chicken health to a wider audience as travelling puppeteers often visit even remote villages. In particular children who are often looking after chickens in a village household could be reached via this method. However, within the time frame of the project it was not possible to deliver a puppet play on village chicken health.

Communication, defined as the sharing of ideas and information, forms a large part of the extension worker’s job. Passing on ideas, advice and information to influence the decisions of farmers is a bi-directional process in which the extension worker has to listen carefully to the farmers’ needs. Farmers might be reluctant to engage in innovations as they might be perceived as too risky (Berdegue 1992). Thus it is necessary to explore farmers’ perceptions in a close collaboration between different disciplines such as veterinary science, social science, anthropology and agricultural science (Forno 1999; Swanson et al. 1997). We believe that epidemiological survey methodology and participatory approaches are important not only to explore current perceptions, attitudes and practices, but also traditional beliefs. Therefore a combination of quantitative and qualitative survey methods is essential to understand what extension message might work or won’t work for a farmer and/or for a village or community under the current conditions.

The spoken word is a key communication tool, but, whether the person delivering the extension message is speaking to a large village meeting or is discussing a problem in the field with a group of farmers, its impact and effectiveness can be greatly increased by the use of suitable audio-visual aids (Oakley and Garforth 1985). Sophisticated audio-visual aids might require electricity and complex equipment such as projectors or televisions, which are often not available in villages of low income countries. Simple aids that can be made locally such as flip charts have several advantages over audio-visual equipment: they do not require a power source, they do not cost much to produce and they can be made to suit the precise needs of the farmers.

Often epidemiological research studies provide a variety of outcomes, but identifying the most important messages to be communicated to farmers can be a challenge. Interventions will only be acceptable by the farmer, if they satisfy the farmer’s need; they have to be technically, economically and ecologically sound. Some needs perceived by farmers might be inappropriate (e.g. upgrading domestic village chicken breeds with exotic breeds that perform poorly in the same scavenging environment) and this has to be carefully discussed with farmers. Also commercial operations will have different constraints than small-scale producers, and preventive messages might not be applicable to both enterprise types in the same way (e.g. fencing-off areas for scavenging village chickens to improve biosecurity might not be feasible for small-scale family poultry farmers).

Sumberg (2005) indicated that research outputs that are not used by farmers are, by definition, not relevant to farmers, and the reasons for this ‘irrelevance’ must be further examined. Hence the adoption process is the procedure in which farmers choose or choose not to use new ideas (or interventions) on their farms (Luukkainen 2012). It is a process that involves gaining new information (usually provided by the extension advisor), a development of interest by the farmer in which he/she changes their attitudes towards the idea, an evaluation and decision making process in which the farmers decides to use or not use the idea, a trial and implementation phase in which the idea is tested, and finally the adoption and confirmation phase, in which the farmer decides if the new idea is preferable over the old methods (Luukkainen 2012). What drives farmers to change or not to change farming practices depends on factors associated with implementation of the idea (e.g. costs and saved expenses, marketing opportunities for the additional product produced, time commitment for implementing the new idea), characteristics of the individual (e.g. age, education level, occupation), community and ecological variables (e.g. disease situation in the village or region, actions that neighbours or relatives implement related to the new idea) and larger social, administrative and political variables (e.g. government, pricing and trade policies) (Leeuwis 2004). All these variables, including the varying adoption outcomes (e.g. short-term, long-term, and perhaps unintended adoption) should be explored in epidemiological-sociological investigations to evaluate the success of the adoption process. Although farmers were eager to adopt the simple interventions in our project, the long-term adoption could not be accessed within the timeframe of the study. The availability of the intervention equipment was probably the most limiting factor for the successful adoption of our promoted interventions. This included the continued production and availability of the I-2 vaccine manufactured by LBVD and the availability of small packages of chick starter feed for village chicken farmers.

Several economic factors are important to both the scope of the activity and the perception of extension assistance to farmers. The first is the profit opportunity available to farmers who improve productivity by applying new technologies brought to their attention (Swanson et al. 1997). Farmer response to new agricultural innovations is directly related to financial advantage from applying such recommendations. We had shown that management changes applied in the intervention study resulted in increased productivity and greater income for the farmer (Henning et al. 2009, 2013) and this higher income was directly shown in extension messages. A second factor is the existing marketing system. Marketing represents the process by which an individual farm or family produce is transferred from the farm to become a value-laden commodity. Mostly live birds are sold in Myanmar for cash to middle men or they are sold by farmers directly in local village markets. Therefore including the marketing of birds in the extension messages was essential. The third factor relates to the general economic environment in which a nation’s agriculture finds itself. The more prosperous agriculture is, the less interest there may be in seeking assistance from an extension service. Myanmar is an agriculture dominated economy and the income per capita is one of the lowest in the region. Hence we experienced very strong interest by village chicken farmers to learn about methods to improve village chicken productivity.

In addition to the factors described previously, we identified a number of specific variables that can influence the success or otherwise of veterinary extension programs in small-scale agricultural settings.

### Role of women

Specific livestock species are often managed by a specific gender in a farmer’s family. In the baseline survey we identified that in 78% of our study households, women were the people working with chickens (Henning et al. 2007). Therefore we actively encouraged the participation of women in the farmer meetings.

### Level of illiteracy

Extension services must operate at the local community level and must address the levels of literacy and education as they affect extension directly (Bagnol 2012). As most village chicken farmers in our project areas were illiterate we used pictures and cartoons rather than complicated written explanations in our extension messages.

### Suitability of interventions to farming conditions

Sufficient knowledge and understanding of the farming system is essential to make it sustainable. We used household surveys to identify farmers’ circumstances in the target area (Henning et al. 2007), then new technologies/intervention strategies were planned, designed and followed by on-farm testing for a period of one year (Henning et al. 2009). In transferring new technology, it is important to understand a farmer’s own evaluation of its performance during early adoption. Feedback from farmers on the research methodology used and on equipment promoted was collected and recommendations given by farmers during pilot-testing and extension workshops were followed up. For instance creep feeders for chick feeding were modified based on the advice given by farmers early in the project.

### Farmers’ resources to adopt interventions

A serious limitation in rural development is the lack of available capital on which to develop a base of economic activity (Swanson et al. 1997). For successful adoption, interventions promoted have to be affordable for farmers. In our project most village chicken farmers could afford the equipment promoted. However, in the beginning of our extension program farmers were able to purchase chick management equipment from the project at subsidized prices to encourage its use. If larger investments are required to adopt interventions, credit opportunities for farmers need to be explored.

### Farmers’ resistance to new technology

Farmers are usually reluctant to accept changes. Methods to reduce resistance include a) encouraging farmers to feel that interventions are partly their own and not devised by outsiders, b) providing support from village leaders or from influential people to farmers, c) ensuring that proposed changes reduce rather than increase the farmers’ burdens and have practical or economic benefits, d) supporting a group consensus on adoption of a new practice and, e) providing realistic and convincing demonstrations in the interventions used. In our project, for example, farmers with positive experience in the use of the interventions were invited to farmer meetings to share their knowledge and experience with other farmers.

### Availability of extension resources

If trainers cannot confidentially support and explain the interventions promoted, farmers will lose interest in the extension program. In our project, training workshops were conducted with LBVD veterinarians on the delivery of extension messages. There were no official livestock extension services in Myanmar during the period of our project. The only existing extension service in Myanmar was the Myanmar Agricultural Service which focussed on extension messages for crop production. Therefore LBVD township veterinarians, who usually only advise smallholder livestock producers on livestock diseases were trained and actively engaged in delivering the projects’ extension messages and became ‘extension workers’ in this project.

### Mobility of farmers to attend extension workshop

Poor farmers may be unable to travel to meeting places far from the village. In particular women, who are involved in the daily food preparation for the household are often unable to travel. Therefore all farmer meetings were conducted in the farmer’s villages in locations easily accessible by farmers (e.g. monasteries).

Extension programs can be evaluated by focusing on (1) inputs, (2) activities, (3) participation, (4) reactions, (5) individual change, (6) organizational change, (7) community change, and (8) national change, but rarely can all of these areas be covered in an assessment (Swanson et al. 1997). Extension effectiveness may also be determined by the number and the regularity of training workshops held with farmers and the number of farmers’ trained (Agbarevo 2013) and in this context we were able to reach a large number of farmers in a wide geographical area with our extension program. Overall, we experienced that extension work can be more challenging than research. We believe that preventive veterinary training and extension programs generally require more time than research studies, in particular when the long-term effectiveness and impacts of extension messages delivered need to be evaluated.

## Conclusions

In summary appropriate communication of epidemiological research study results combined with a strong policy commitment are required to succeed in meeting the needs of resource-poor farmers. Considering the Bayesian paradigm, local prior knowledge should be considered in the development of any interventions or extension messages. In addition it is essential to evaluate the success of an extension program by measuring the uptake of messages and to ensure that the extension process has become self-sustainable. The commonly used paradigm of ‘One health, One world’ can also be used to characterise the beneficiaries of a research project: a researcher has not only the responsibility to publish outcomes of a research program and to discuss these outcomes with other scientists or risk managers, but also has the responsibility to communicate these research findings in an understandable manner to the people who provided the data.

## Authors’ original submitted files for images

Below are the links to the authors’ original submitted files for images. Authors’ original file for figure 1 Authors’ original file for figure 2 Authors’ original file for figure 3
