# Supplementary figures and images for: Interdisciplinary communication of infectious disease research – translating complex epidemiological findings into understandable messages for village chicken farmers in Myanmar
Source: Springerplus. 2014 Dec 11;3:726. doi: 10.1186/2193-1801-3-726 (PMC4320238; doi:10.1186/2193-1801-3-726)

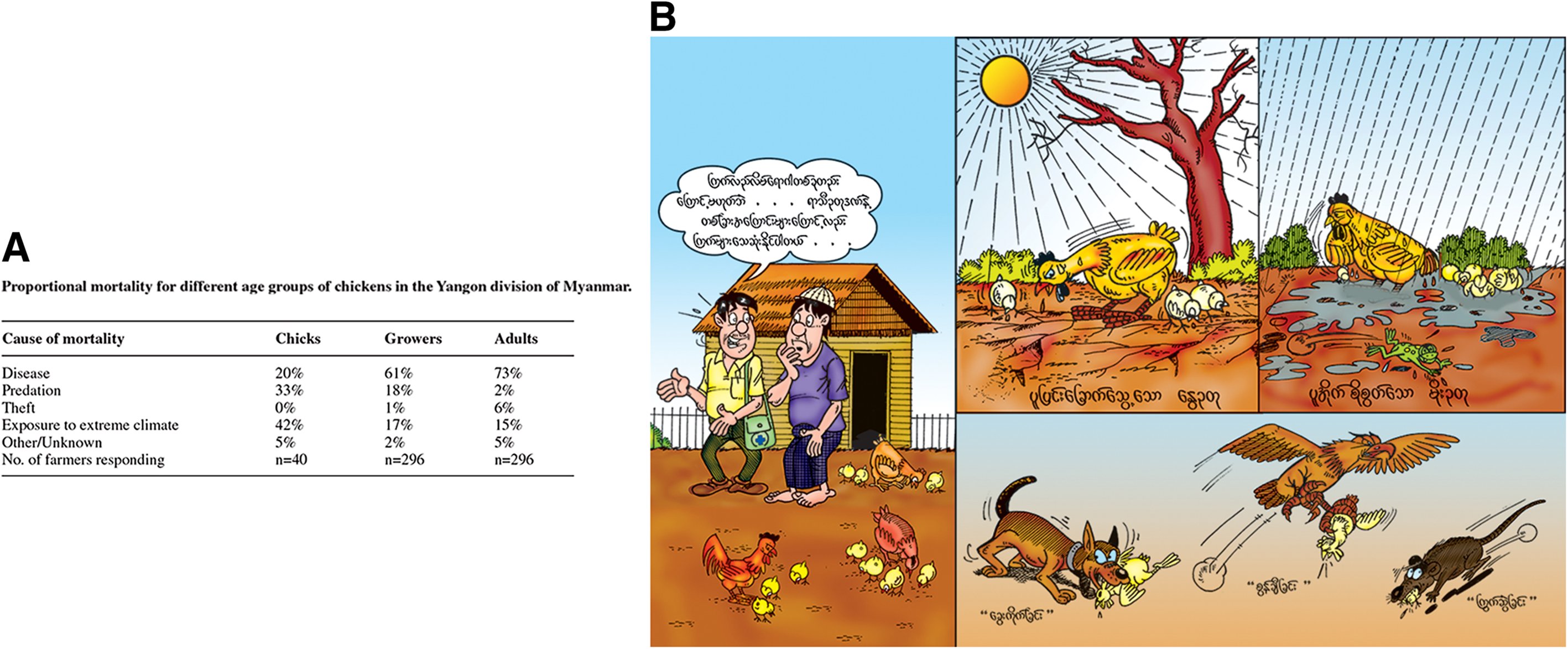

Supplement: Supplementary file 1 — Authors’ original file for figure 1 [file 40064_2014_1487_MOESM1_ESM.tiff]

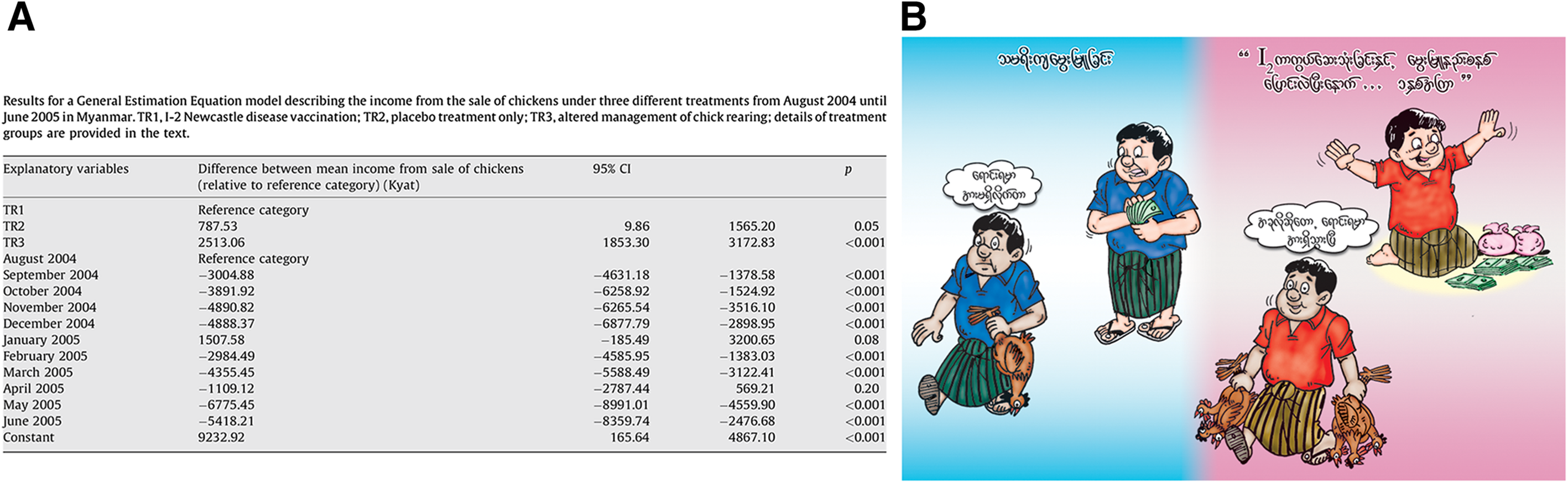

Supplement: Supplementary file 2 — Authors’ original file for figure 2 [file 40064_2014_1487_MOESM2_ESM.tiff]

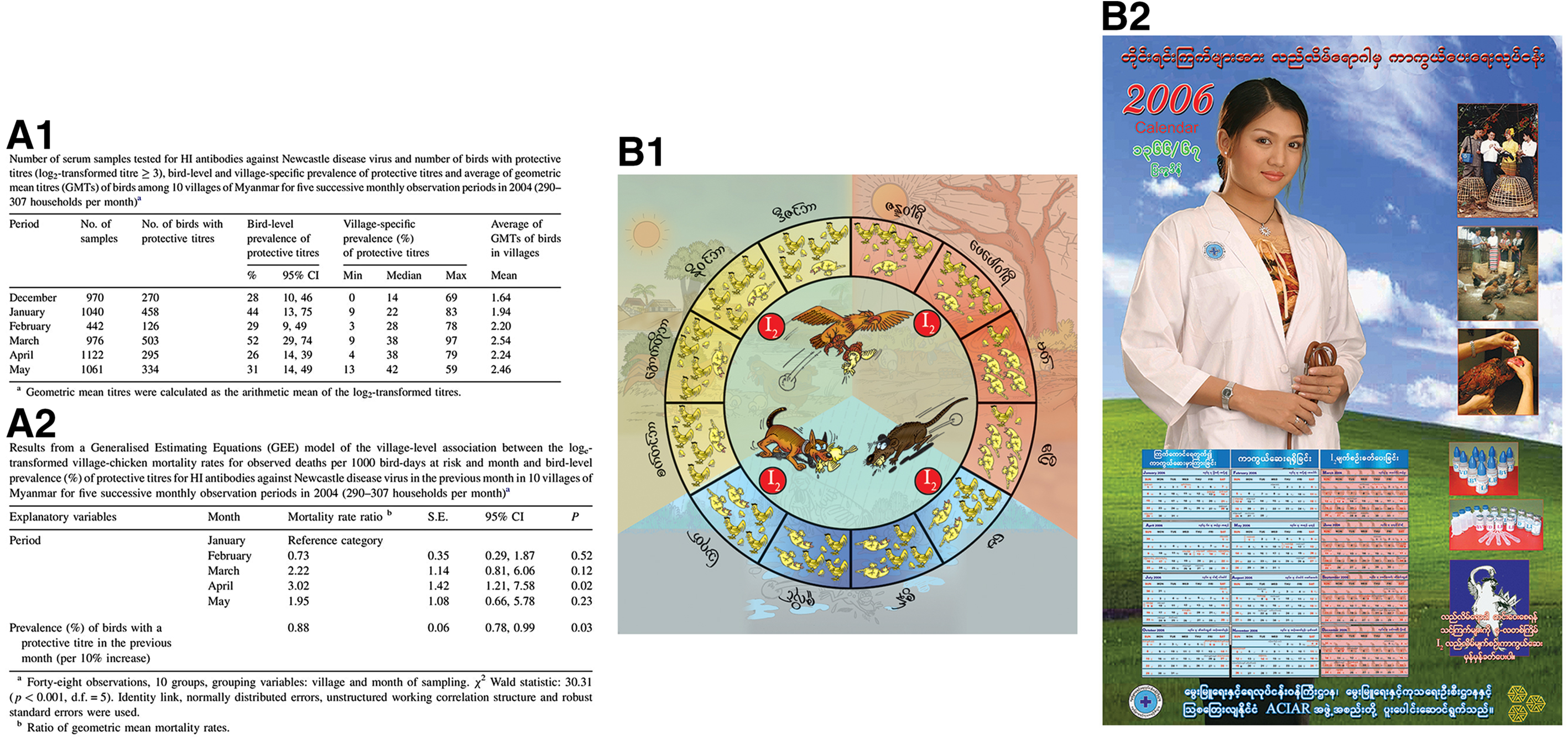

Supplement: Supplementary file 3 — Authors’ original file for figure 3 [file 40064_2014_1487_MOESM3_ESM.tiff]
